# *TANGO2*: A Rare but Important Mutation

**DOI:** 10.19102/icrm.2024.15054

**Published:** 2024-05-15

**Authors:** Benjamin Walters, Nathan McConkey, Jason R. Imundo

**Affiliations:** 1 Pennsylvania State University School of Medicine, Hershey, PA, USA; 2 Heart and Vascular Institute, Penn State Milton S. Hershey Medical Center, Hershey, PA, USA; 3 Division of Pediatric Cardiology, Penn State Health Children’s Hospital, Hershey, PA, USA

**Keywords:** Hypothyroidism, long QT interval, recalcitrant ventricular arrhythmias, rhabdomyolysis, *TANGO2*

## Abstract

We report the case of a 7-year-old boy who presented with post-viral myositis, rhabdomyolysis, and hepatitis, who was later readmitted due to a seizure-like activity and ultimately found to have episodes of recalcitrant polymorphic ventricular tachycardia secondary to simultaneous QT prolongation and severe hypothyroidism. Temporary transvenous atrial pacing was successful at controlling the ventricular arrhythmias in the intensive care unit. With levothyroxine therapy and cessation of QT-prolonging medications, the corrected QT (QTc) normalized. A comprehensive arrhythmia panel identified a pathogenic mutation in *KCNQ1*, consistent with long QT syndrome (LQTS) type 1. After the patient experienced progressive neurodegeneration and seizures, he was referred to a genetics clinic to rule out genetic epilepsy. On the epilepsy panel of genetic testing, he was found to have two pathogenic variants in *TANGO2*. *TANGO2* deficiency explains the initial presentation of myositis, rhabdomyolysis, hypothyroidism, and life-threatening arrhythmias surrounding a viral illness more so than the initial diagnosis of mere LQTS. However, the *TANGO2* gene is not included in most comprehensive arrhythmia and cardiomyopathy panels. *TANGO2* deficiency is a rare condition that often presents with arrhythmias but may be unfamiliar to many cardiologists and electrophysiologists. This case describes management strategies and caveats, which could aid in the successful diagnosis and treatment of *TANGO2* deficiency at the time of presentation.

## Case presentation

A 7-year-old boy presented with lower-lobe pneumonia in the setting of influenza B, and he was treated with oseltamivir and amoxicillin. After failing to improve and developing unexplained transaminitis, he was transferred to our hospital and diagnosed with acute hepatitis, post-influenza B myositis, and rhabdomyolysis with unremitting pain and immobility. He had a normal echocardiogram, but an electrocardiogram (ECG) was not performed. He was discharged to a rehabilitation hospital but presented 2 weeks later to our emergency department (ED) and was readmitted to our children’s hospital due to new-onset seizure-like activity associated with cyanosis and a postictal state. His past medical history was significant for transient episodes of slurred speech, weakness, and an abnormal gait at 6 years of age. A workup at that time demonstrated a normal electroencephalogram and ECG showing normal sinus rhythm with a corrected QT (QTc) of 437 ms, non-specific U-waves, and early repolarization. The patient’s family history was significant for a paternal aunt who passed away suddenly at 25 years of age from an unknown cause.

**Figure 1:**
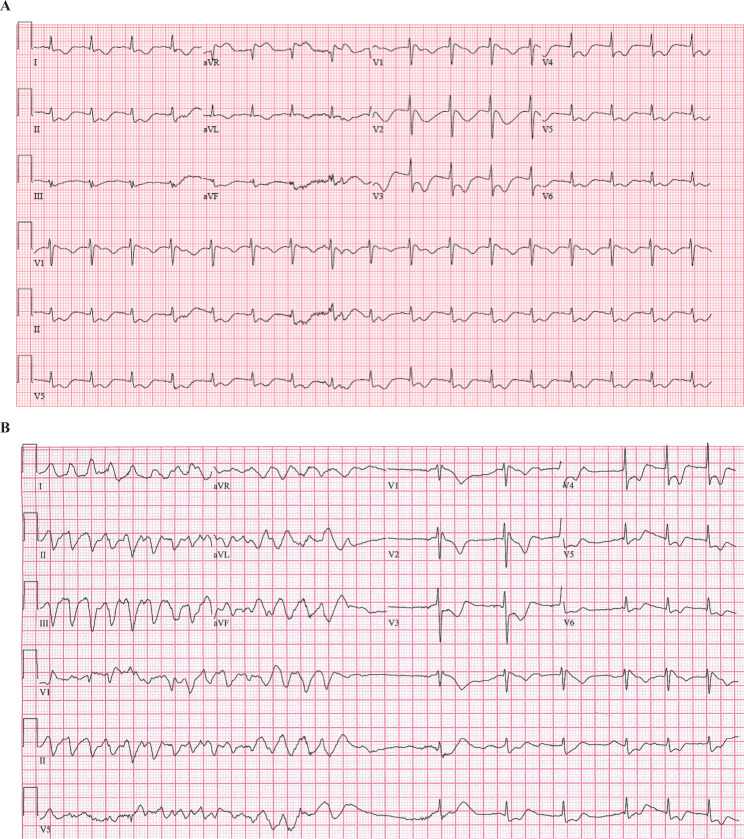
**A:** Initial electrocardiogram showing a normal sinus rhythm with a prolonged corrected QT interval of 578 ms, diffuse ST-segment depression, and grossly abnormal T-waves in inferolateral leads. **B:** Spontaneously resolving pulseless polymorphic ventricular tachycardia.

On admission, his thyroid-stimulating hormone level was >100 UIU/mL (normal, 0.7–6.4 UIU/mL) and his thyroxine (T4) and triiodothyronine (T3) levels were 1.2 μg/dL (normal, 6.4–13.3 μg/dL) and 1.7 pg/mL (normal, 2.8–5.3 pg/mL), respectively. On hospital day 2, the patient had a witnessed seizure-like event and was immediately placed on telemetry due to concern for arrhythmia. A second event occurred shortly after providing electrographic evidence of polymorphic ventricular tachycardia requiring brief cardiopulmonary resuscitation with return of spontaneous circulation. An emergent echocardiogram demonstrated low-normal left ventricular (LV) function and a structurally normal anatomy. An ECG from admission revealed a grossly abnormal T-wave morphology, a QTc of 578 ms, and diffuse ST-segment depression **(Figure 1A)**.

**Figure 2:**
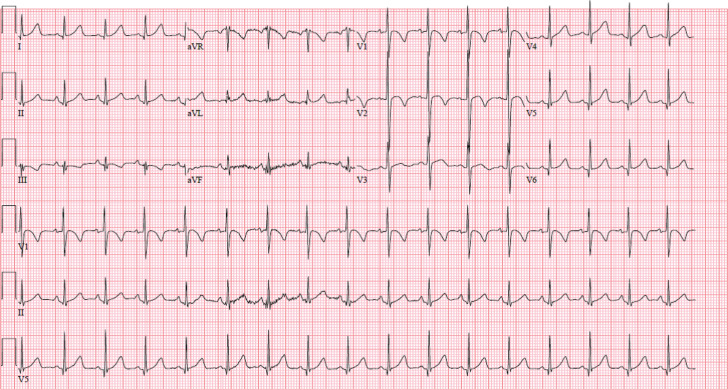
Electrocardiogram taken 3 months post-discharge on β-blocker therapy showing sinus rhythm and a normalized corrected QT interval of 436 ms.

The patient continued to have episodes of polymorphic ventricular tachycardia and ventricular fibrillation **(Figure 1B)**, occasionally requiring defibrillation before converting. Intravenous (IV) esmolol was unsuccessful at suppressing episodes of polymorphic ventricular tachycardia. Secondary to the episodes of polymorphic ventricular tachycardia appearing to be pause-dependent, temporary transvenous atrial and ventricular pacing wires were placed. Atrial pacing successfully suppressed further episodes of ventricular tachycardia. On hospital day 3, an echocardiogram revealed a dilated left ventricle with dramatically worsening LV function compared to that recorded during a prior study. Throughout his hospital course, he continued to have intermittent wide-complex rhythms without hemodynamic instability. His free T4 level reached therapeutic levels by hospital day 10, and, after increasing levothyroxine to 100 mg/day, his free T3 level reached a low-normal level of 2.9 pg/mL. Despite this, on hospital day 11, he developed widening of the QRS and hypotension, followed by cardiac arrest. Return of spontaneous circulation was achieved after 30 min. He was placed on venoarterial extracorporeal membrane oxygenation (ECMO) and started on intravenous T3, after which he slowly began to improve. He was eventually weaned off ECMO and all pressors. His QTc normalized; however, when his longstanding intravenous pain regimen was transitioned to methadone, he again developed QT prolongation, which normalized (QTc of 436 ms) after discontinuation of the drug **(Figure 2)**.

Electrocardiographic evidence of a prolonged QTc, abnormal T-wave morphology, and ventricular arrhythmias supported the initial diagnosis of long QT syndrome (LQTS). He underwent genetic testing, including a comprehensive arrhythmia and cardiomyopathy panel, in April 2013 through GeneDx (Gaithersburg, MD, USA), which confirmed a disease-causing class I variant in *KCNQ1 (p.Ala341Gly)*, a gene with a known association with LQTS type 1. We had been following the patient for 8 years, and he had remained asymptomatic on levothyroxine and nadolol. He has had normal QT measurements on follow-up ECGs, no known metabolic crises, rhabdomyolysis, or seizure activity until October 2022, when he began experiencing seizures and worsening neurodegeneration, including cognitive impairments and ataxia. He was referred for genetic testing to rule out genetic epilepsy; an epilepsy panel from Invitae (San Francisco, CA, USA) was performed in December 2022, and studies revealed two pathogenic variants (deletion, exons 3–9, homozygous; c.57-?_*2502+?Del) in *TANGO2*, a gene associated with metabolic encephalopathy, rhabdomyolysis, hypothyroidism, and prolonged QT intervals and arrhythmias during metabolic crises, explaining his initial symptomatology and presentation.^1^

All procedures mentioned in this case report, which did not involve human experimentation, were performed in accordance with the ethical standards of the United States of America guidelines on human treatment. Verbal consent was obtained for this work.

## Discussion

*TANGO2* deficiency is a rare, genetic, metabolic disorder characterized by developmental delay, gait incoordination, hypothyroidism, and recurrent crises with associated metabolic derangements, myositis, rhabdomyolysis, and severe cardiac tachyarrhythmias.^1^ *TANGO2* deficiency was initially described in 2016 as a biallelic loss-of-function mutation in the *TANGO2* gene located on chromosome 22q11.2, resulting in childhood metabolic crises induced by a catabolic metabolic state, such as an infection.^2^ Since the initial 2016 report, *TANGO2* deficiency has increased in volume in the literature, with 77 cases reported to date.^2^ The function of the *TANGO2* gene is still not clear; however, data suggest multiple potential functions of the TANGO2 protein, including a role in fusion of the Golgi apparatus with the endoplasmic reticulum,^3^ and mitochondrial structure and function.^4^ *TANGO2* deficiency is thought to induce dysfunction in lipid biosynthesis that is exacerbated under conditions that stress the body.^5^

Patients with *TANGO2* deficiency tend to have similar initial presentations, commonly presenting with metabolic crisis precipitated by an infection or other catabolic state, and patients will usually have a history or current symptoms of psychomotor impairment characterized by ambulatory dysfunction and hypotonia as well as signs of neurodevelopmental decline.^6^ The metabolic crises that most previous patients had during their workup included similar findings of rhabdomyolysis, seizures, ECG abnormalities including prolonged QT intervals and/or type I Brugada pattern, and severe arrhythmias including potentially life-threatening recalcitrant ventricular tachycardia.^1^ There are no current diagnostic criteria for *TANGO2* deficiency to date, and the diagnosis is made using molecular genetic testing. Most commonly, the disease-causing mutation is a deletion of exons 3–9 on chromosome 22 that results in a non-functional protein.^4^ Other reports suggest that pathogenic variants include splice site, missense, and nonsense variants as well as single-exon deletion that can precipitate as *TANGO2* deficiency and may cause mitochondrial dysfunction.^4,7^

The patient’s initial diagnosis of LQTS type 1 explained some, but not all, of his symptoms. This initial diagnosis was especially supported by a confirmed mutation in *KCNQ1* found on a comprehensive arrhythmia panel. This mutation was initially classified as a disease-causing variant at the time of his diagnosis, and it continues to be currently classified as a *likely* pathogenic variant (*p.Ala341Gly*, Invitae 2022). For our patient, we started an initial regimen of β-blocker therapy for LQTS, but withheld implantable cardioverter-defibrillator placement unless he failed optimal medical management.^8^ It was only after 8 years of follow-up that he presented with worsening neurologic manifestations, at which point genetic studies for epilepsy revealed the *TANGO2* variant. As our patient was noted to have QTc prolongation while taking prescribed methadone and based on his likely pathogenic variant in *KCNQ1*, we elected to continue his nadolol. Gene-specific testing of the immediate family was performed, and this *KCNQ1* mutation was found in the patient’s mother, who was reported to be phenotype-negative on cardiology follow-up. The extended family did not receive gene-specific testing secondary to social and geographic constraints related to living outside of the United States. The patient’s father did not have this mutation; therefore, we could deduce that the paternal aunt who passed away at the age of 25 years also did not have this *KCNQ1* mutation.

*TANGO2* deficiency has distinct differences from LQTS in the way patients and their families are counseled and managed; however, given that the first description of the former disorder was published only in 2016, many physicians may be unfamiliar with the condition, and the diagnosis may elude many cardiologists and electrophysiologists. There is currently no cure or published management guidelines for this condition; however, daily supplementation with B vitamins is thought to reduce the risk of metabolic crises and resulting cardiac arrhythmias, and continued nutritional support with multivitamin and/or B-complex supplementation inclusive of vitamins B5 and B9 is believed to be helpful.^1,9^ The remainder of therapy remains supportive.^1^ If they do arise, treatment of *TANGO2* cardiac crises includes intravenous magnesium, isoproterenol, and atrial pacing.^10^ Manifestations of cardiac involvement in *TANGO2* deficiency typically present only during times of crisis. They include ventricular tachycardia that may be recalcitrant to anti-arrhythmic medications, cardiac arrest, prolonged QT intervals and/or type I Brugada pattern, hemodynamic instability, and unexplained cardiovascular collapse. Many patients require ECMO during periods of substantial instability.

This is not the first case report to describe this phenotypic presentation in a patient who was ultimately diagnosed with *TANGO2* deficiency, and this is the second reported case found on genetic screening after an initial diagnosis and management of suspected LQTS.^2^ However, a unique factor in our case is that our patient responded well to β-blocker therapy alone for a period of 8 years prior to the eventual diagnosis of *TANGO2* deficiency, at which time management specific to this mutation was implemented. He is currently managed on ubiquinol, vitamin B6 (pyridoxine), vitamin B5 (pantothenic acid), vitamin B100, vitamin D, and a multivitamin. The patient and his family also had an emergency action plan provided to them in writing, which was also placed in the patient’s chart to alert the ED that the patient should bypass ED triage and waiting lines and have an intravenous line placed, labs sent, and 150% the maintenance fluid infusion rate provided to diagnose and lessen the chance of having a metabolic crisis, respectively. We have recommended telemetry monitoring and a 12-lead ECG at the time of presentation to the ED.

Our patient’s clinical findings are similar to those of other patients with *TANGO2* deficiency; however, he has had a much slower rate of progression of neurodegeneration and an overall improved clinical course compared to other patients with *TANGO2* deficiency described in the literature. He is now 18 years old and in the 11th grade, and he is in some regular classes and some classes that are given through an individualized education program. He has some difficulty with speech, spelling, and doing calculations with complex numbers. He exercises and can run 10 laps of 100 yards/lap with a gait disturbance, and he enjoys playing recreational soccer in a defense position. Interestingly, the patient’s family started him on a multivitamin containing B-complex vitamins immediately after his cardiac arrest. It is possible that this intervention may be responsible for our patient’s clinical course, which is less severe than most other reported patients with *TANGO2* deficiency. We believe that our patient’s diagnosis of *TANGO2* deficiency rather than LQTS is responsible for his initial presentation described in this case report. This is supported by the improvement in his arrhythmias with temporary transvenous atrial pacing and his lack of response to intravenous esmolol. However, knowledge of the *KCNQ1* variant is important for future monitoring, recommendations, and treatment. We have made the decision to continue his nadolol given that he has been tolerating this medication very well for many years, his finding of the likely pathogenic *KCNQ1* variant, and his noted QTc prolongation at the time he was placed on a medication known to prolong the QT. This report provides compelling evidence for inclusion of the *TANGO2* gene mutation into the comprehensive arrhythmia and cardiomyopathy genetic panels, where it is not presently included, due to the potential change in management and counseling that may have been provided to the patient and his family if it was diagnosed at initial genetic testing.

## Conclusion

*TANGO2* deficiency is a rare but important disease that should be considered in patients presenting in an acute metabolic crisis with life-threatening recalcitrant ventricular arrhythmias. Early recognition of the spectrum of symptoms, which include not just arrhythmias, but also weakness, slurred speech, gait abnormality, rhabdomyolysis, and hypothyroidism, will help facilitate early diagnosis and appropriate treatment for these patients. *TANGO2* genetic testing is included in many comprehensive epilepsy, neuromuscular, encephalopathy, and metabolic panels, but, at the time this manuscript was written, it is not included in most comprehensive arrhythmia/cardiomyopathy panels. This case emphasizes the importance of regular reassessment of a clinical picture, especially when there are aspects of a case that do not make sense, which allows for an opportunity to adjust diagnoses and management plans.

## References

[1] Miyake CY, Burrage L, Glinton K,  Adam MP, Feldman J, Mirzaa GM (2018). GeneReviews^®^ [Internet].

[2] Gomes SA, Laranjo SDS, Trigo C, Pinto FF (2023). The TANGO2 disease and the therapeutic challenge of acute arrhythmia management: a case report. Eur Heart J Case Rep.

[3] Lalani SR, Liu P, Rosenfeld JA (2016). Recurrent muscle weakness with rhabdomyolysis, metabolic crises, and cardiac arrhythmia due to bi-allelic TANGO2 mutations. Am J Human Genet.

[4] Heiman P, Mohsen AW, Karunanidhi A (2022). Mitochondrial dysfunction associated with TANGO2 deficiency. Sci Rep.

[5] Asadi P, Milev MP, Saint-Dic D, Gamberi C, Sacher M (2023). Vitamin B5, a coenzyme A precursor, rescues TANGO2 deficiency disease-associated defects in Drosophila and human cells. J Inherit Metab Dis.

[6] Mingirulli N, Pyle A, Hathazi D (2020). Clinical presentation and proteomic signature of patients with TANGO2 mutations. J Inherit Metab Dis.

[7] Dines JN, Golden-Grant K, Lacroix A (2019). TANGO2: expanding the clinical phenotype and spectrum of pathogenic variants. Genet Med.

[8] Cho Y (2016). Management of patients with long QT syndrome. Korean Circ J.

[9] Sandkuhler SE, Zhang L, Meisner JK (2023). B-complex vitamins for patients with TANGO2-deficiency disorder. J Inherit Metab Dis.

[10] Miyake CY, Lay EJ, Beach CM (2022). Cardiac crises: cardiac arrhythmias and cardiomyopathy during TANGO2 deficiency related metabolic crises. Heart Rhythm.

